# ﻿*Parartemiopsisshangrilaensis*, a new species of fairy shrimp (Branchiopoda, Anostraca) from Yunnan, with a key to the Chirocephalidae of China

**DOI:** 10.3897/zookeys.1168.104005

**Published:** 2023-07-04

**Authors:** Shu-Sen Shu, Xiao-Yong Chen, D. Christopher Rogers, Laorsri Sanoamuang

**Affiliations:** 1 Southeast Asia Biodiversity Research Institute, Chinese Academy of Sciences, Yezin, Nay Pyi Taw 05282, Myanmar; 2 State Key Laboratory of Genetic Resources and Evolution and Yunnan Key Laboratory of Biodiversity Conservation of Gaoligong Mountain, Kunming Institute of Zoology, Chinese Academy of Sciences, Kunming, Yunnan, 650201, China; 3 Applied Taxonomic Research Center, Khon Kaen University, Khon Kaen 40002, Thailand; 4 Kansas Biological Survey, and The Natural History Museum (Biodiversity Institute), The University of Kansas, Lawrence, Kansas 66047-3759, USA; 5 International College, Khon Kaen University, Khon Kaen 40002, Thailand

**Keywords:** Asia, crustacean, diversity, freshwater, taxonomy, Tibetan Plateau

## Abstract

The fairy shrimp genus *Parartemiopsis* Rogers, 2005 currently contains a single species reported from Russia and Mongolia. In 2013, an unidentified *Parartemiopsis* population was reported from the eastern margin of the Tibetan Plateau in China’s Yunnan Province, from Patatson National Park in Shangri-La County. Here, we describe the Chinese populations as a new species, *Parartemiopsisshangrilaensis***sp. nov.** This new species is distinguished from its congener, *P.longicornis* (Smirnov, 1930), by the form of the male second antennae and the gonopod. The discovery of *P.shangrilaensis***sp. nov.** extends the known distribution of the genus, and more *Parartemiopsis* species may be found in the future. We present a key to the genera and species of Chirocephalidae in China as an aid to future research.

## ﻿Introduction

Chirocephalidae is the second largest anostracan family in terms of numbers of species, but it contains the most genera (Belk and Brtek 1995; Rogers et al. 2013). The family contains nine genera, with all but one species (*Chirocephalushardingi* Brtek, 1965 from Bali) distributed entirely in the Holarctic. *Parartemiopsis* Rogers, 2005 was erected (Rogers 2005, 2006) to contain *Chirocephaluslongicornis* (Smirnov, 1930). Rogers (2005, 2006) demonstrated that this species warranted a separate genus and was morphologically closer to *Artemiopsis* Sars, 1897 than to *Chirocephalus* Prévost, 1803.

An unidentified *Parartemiopsis* population was reported from Yunnan Province (Shu et al. 2013) in a list of crustaceans from Patatson National Park. More material was collected and is described here as a new species. A key to the species in the genus is presented, along with a key to the Chirocephalidae of China. It is our hope that this key will further future studies on the Chirocephalidae of this region.

## ﻿Materials and methods

Specimens were collected from Patatson National Park in 2012 and 2019 using a dip net. Specimens were preserved on site in 95% alcohol. Specimens were examined in the laboratory under a stereomicroscope (Zeiss Stemi 508 and Wild M8) and a compound microscope (Olympus CX31). For scanning electron microscopy (SEM), specific structures were dissected from individual specimens and put into a 2.5% glutaraldehyde solution for 24 h, washed for 30 min three times with 0.01 M phosphate buffered saline, transferred to a 1% osmic acid solution for 2 h, then washed three times for 30 min each with 0.01 M phosphate buffered saline. Next, the material was treated in an ethanol gradient dehydration series at 30%, 50%, 70%, 80%, 90%, 95%, and 100% for 20 min. The specimens were then put into tertiary butyl alcohol at 40 °C for 2 h and frozen at −20 °C. The material was then freeze-dried in a JFD-310 freeze dryer. Later, the specimens were mounted on stubs and coated with gold in a JFC-1600 sputter coater. SEM images were taken using a TM 1000 Hitachi scanning electron microscope at 10 kV. The pictures were collaged in Photoshop CS.

Terminology follows Rogers (2005, 2006) and Rogers and Soufi (2015). Examined specimens are deposited in the Kunming Natural History Museum, the Kunming Institute of Zoology (KIZ), Chinese Academy of Sciences.

## ﻿Results

### ﻿ Parartemiopsis

Taxon classificationAnimaliaAnostracaChirocephalidae

Rogers, 2005

B759D044-A5A2-506D-9CD7-EE1EDF100F02

Parartemiopsis Rogers 2006, 2013.
= Pristicephalus pro partim in Smirnov 1930; Brtek 1958.
= Chirocephalus pro partim in Brtek 1966, 1967, 1997, 2002; Belk and Brtek 1995.

#### Diagnosis.

(modified from Rogers 2006). Male genital segments expanded. Entire gonopod basal portion chitinized. Gonopod basal portion bearing a broad lamellar ventral process, originating anterior to gonopod base. Gonopod laterally with tubercle. Gonopod distal eversible portion not cirriform, bearing a few scattered denticles.

### ﻿ Parartemiopsis shangrilaensis sp. nov.

Taxon classificationAnimaliaAnostracaChirocephalidae

B20C1E6C-9AD7-5C2E-988D-478C57818B6D

https://zoobank.org/5062BFD3-2DC9-4C8F-B3E7-787B458BFCFF

**Figure 1.**
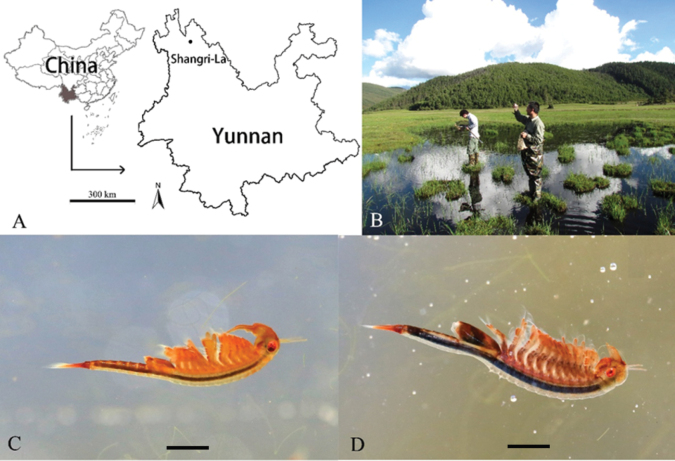
*Parartemiopsisshangrilaensis* sp. nov. **A** type locality in Shangri-La, China (black spot) **B** environment of the type locality and collection of the specimens **C** male, lateral view **D** female, lateral view (photo by W-S Jiang). Scale bars: 2 mm (**C, D**).

**Figure 2.**
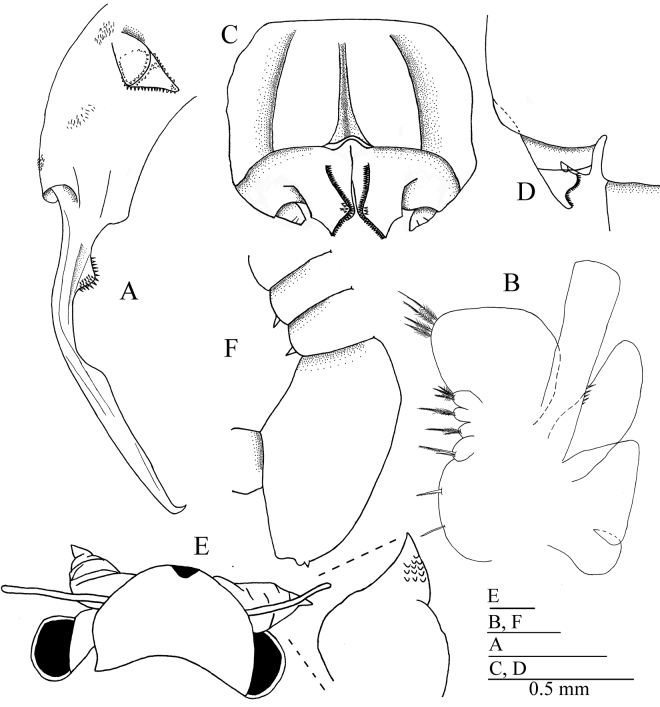
*Parartemiopsisshangrilaensis* sp. nov. **A–D** male **E, F** female **A** second antenna, anterior view **B** thoracopod V, only spines are shown **C** genital segments, ventral view **D** genital segments, lateral view **E** head and detail of second antenna, anterior view **F** thoracic segments X and XI, and genital thoracic segments, lateral view.

**Figure 3.**
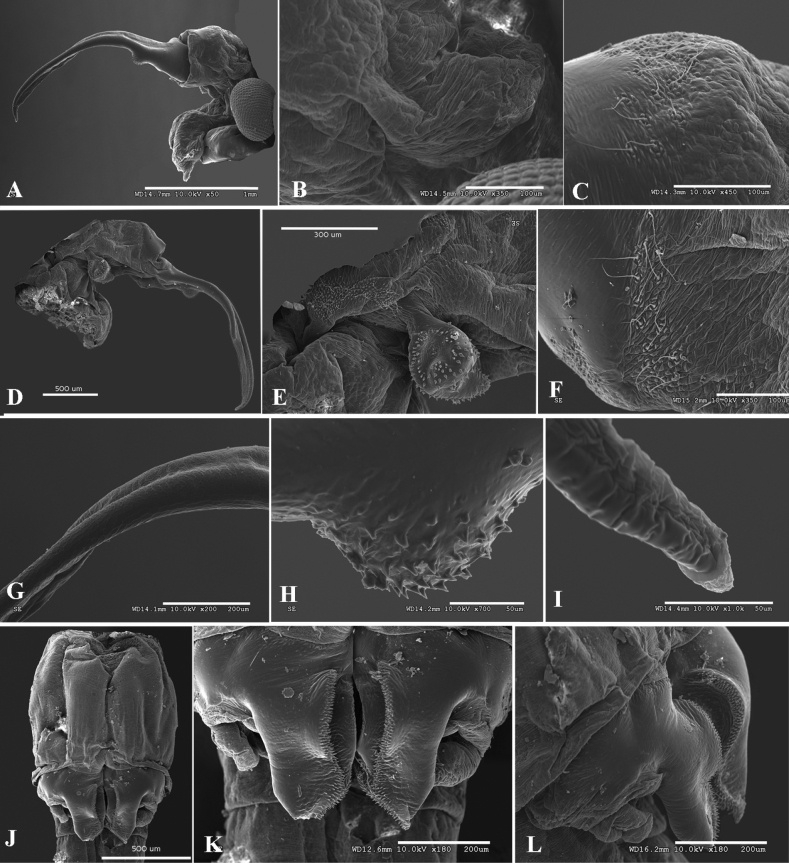
*Parartemiopsisshangrilaensis* sp. nov., male **A** second antenna posteriolateral view **B** second antenna proximal antennomere posterior surface detail **C** second antenna proximal antennomere lateral surface detail and setae **D** second antenna anterior view **E** antennal appendage **F** medial surface detail **G** second antenna distal antennomere detail **H** second antenna distal antennomere medial protuberance **I** second antenna distal antennomere apex **J** genital segments **K** uneverted gonopods, ventral view **L** uneverted gonopods, ventrolateral oblique view.

**Figure 4.**
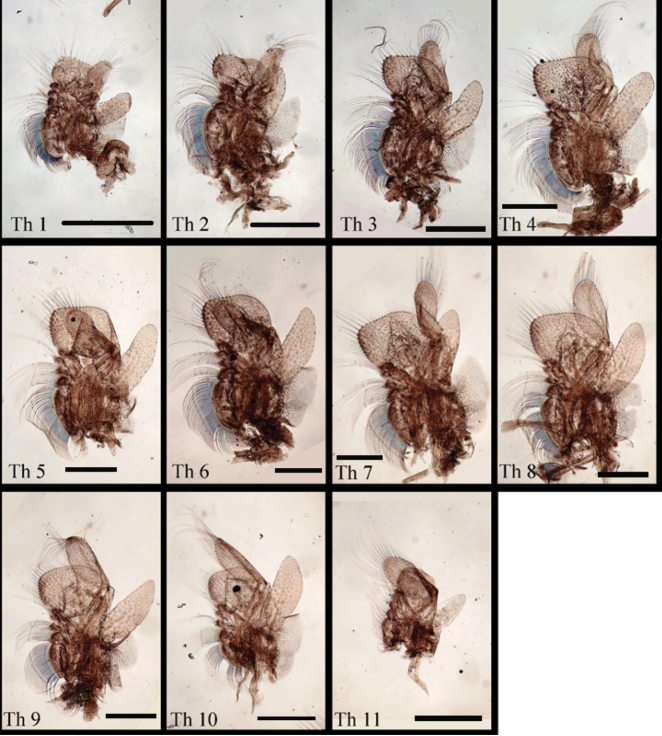
*Parartemiopsisshangrilaensis* sp. nov., thoracopods I–XI. Th = thoracopod. Scale bars: 1 mm.

**Figure 5.**
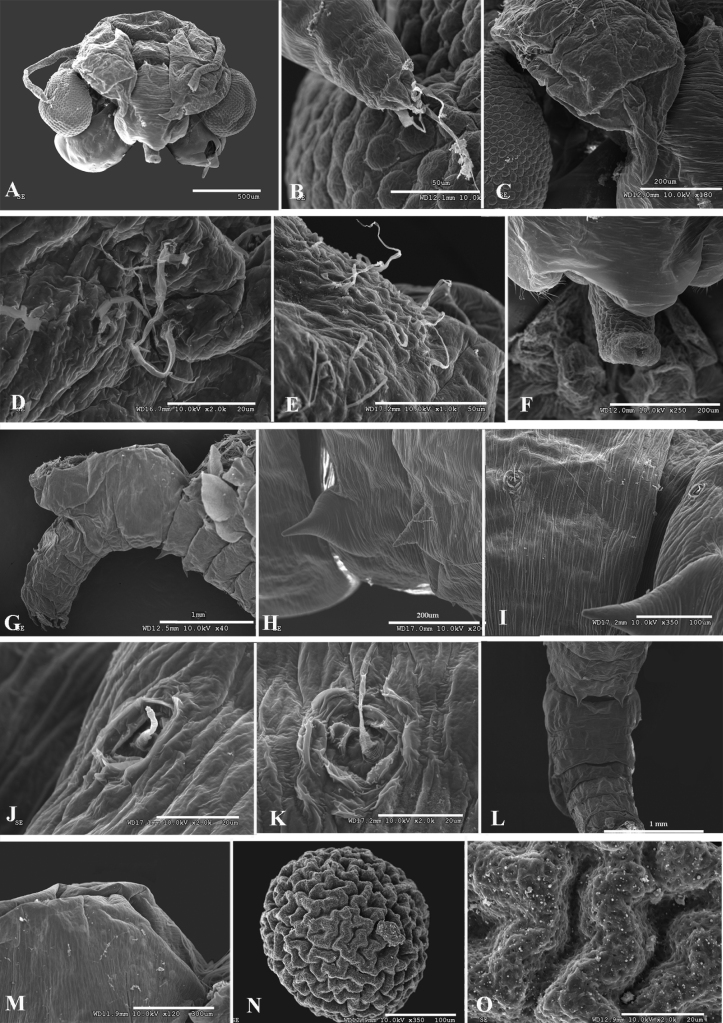
*Parartemiopsisshangrilaensis* sp. nov. female **A** head, ventral view **B** first antenna apex **C** second antenna **D** second antenna proximal surface setae **E** second antenna lateral surface setae **F** labrum apex **G** distal thoracic segments and brood pouch in left lateral view **H** thoracic segments X and XI dorsal spines **I** thoracic segment XI and genital thoracic segment setae **J** thoracic segment XI seta **K** genital thoracic segment seta **L** thoracic segments X, XI and genital thoracic segment in dorsal view **M** brood pouch proximal part **N** egg **O** egg detail.

Figs 1A –D , 2A –F , 3A –L , 4 , 5A –O

= Parartemiopsis sp. Shu et al. 2013.

#### Type materials.

***Holotype***: one male in alcohol, KIZ–CR-2011010. ***Allotype***: one female in alcohol, KIZ–CR-2011011.

#### Type locality.

China: Yunnan Province: Shangri-La County: Patatson National Park: 27°52′2″N, 99°58′58″E, 3670 m a.s.l., a small, shallow temporal pond in Militang meadow; 20 August 2011; Shu S-S, Jiang W-S, and Zhao Y-P leg. Militang meadow is in an alpine valley surrounded by mountains covered in coniferous forest. A small river winds through the valley, which receives runoff from the surrounding slopes. The meadow has a mosaic of wetlands and uplands. This species is so far only known from the type locality.

#### Additional materials examined.

Same data as types: one male, six females; 20 August 2011. Same location data as types: 10 males, 48 females; 16 August 2019; Jiang W-S, leg.

#### Similar species.

*Parartemiopsislongicornis* (Smirnov, 1930).

#### Diagnosis.

Typical for genus. Male antennal appendage lamellar, subtriangular, apically subacute (Fig. 3E). Second antenna proximal antennomere lacking lateral hemispherical bulge. Second antenna distal antennomere medial surface with subproximal rounded protrusion covered in spinulae (Fig. 3H). Genital segment medial lamellar projection with medial longitudinal lines of spinulae elevated on crests (Fig. 3K).

#### Description.

Average length (head anterior margin to telson posterior margin): 7.4 mm (range 6.2–8.7 mm) in males; 7.9 mm (range 6.2–10.2 mm) in females.

***Holotype male*.** Body red or jacinth in life (Fig. 1C). Head typical for genus. Eye plus peduncle length subequal to first antenna length. Eye large, width >0.2× second antennal proximal antennomere medial width. First antenna long, filiform, extending to second antennae distal antennomere midlength, apex with five or six aesthetascs.

Antennal appendage lamellar, directed anteromedially, apex broadly triangular (Figs 2A, 3E). Appendage basal portion inerm, distal portion bearing conical spinulae arranged in two or three marginal rows. Anterior surface with scattered, large, anteriodistally directed spines. Each anterior surface spine conical, 2–3× lateral spinulae.

Second antenna extending to thoracopod IV or V (Fig. 1C). Second antenna proximal antennomere (Fig. 3A) subcylindrical, length nearly 1.5× breadth. Posteriolateral surface with medial patch bearing ~10 setae (Fig. 3B). Lateral surface with rings composed of four or five micropapillae surrounding a single filiform seta (Fig. 3C). Anteromedial surface with denticulae and sparse setae (Fig. 3E). Anterior surface with five or six rows of setae, with groups of scattered micropapillae arranged as on lateral surface (Fig. 3F).

Distal antennomere laterally compressed, length ~1.5× proximal antennomere. Distal antennomere narrowing to medial bend ~70° at proximal third of antennomere length (Figs 2A, 3A, G). Just proximad to bend, medial surface with hemispherical protuberance covered in fine denticulae (Fig. 3A, H). Distal two-thirds parallel-sided with remainder straight to apex which is medially bent ~35° (Fig. 3A). Anterior margin with triangular projection midway between bend and apex. Apex subacute (Fig. 3A, I).

Thoracic segments dorsally smooth (Fig. 1C). Thoracopod I with praeepipodite divided, proximal portion oval, distal portion elongate-oval, both with margins inerm and smooth. Epipodite lamellar, length ~2× width, apex angular. Exopod elongate oval, length < epipodite, proximolateral margin with five spines, remaining margins with plumose setae. Endopod broadly oval, margined with plumose setae. Endite VI broadly rounded, with long plumose marginal setae in two groups, bearing one and two middle submarginal spines, respectively. Endite V semicircular, proximal margin with two spiniform setae, remaining margins with plumose setae. Endites VI and III each with long plumose submarginal setae and three spiniform marginal setae. Endite I + II breadth greater than endites II–VI combined, bearing long plumose submarginal setae and one stout, spiniform marginal seta. Thoracopods II–XI serially homologous (Figs 2B, 4) with respect to thoracopod I, endites III–V margined with two or three spines.

Genital thoracic segments fused, ventrally expanded. Genital segment I (Fig. 3J) smooth, elongate, with paired longitudinal ventromedial ridges, their combined width approximately half segment width. Genital segment I distal margin with bead.

Gonopod rigid basal portion (Figs 2C, D, 3K) with ventromedial longitudinal lamellar process, projecting posteriorly, with medial and lateral margins inerm, submedially with longitudinal arcuate carina margined with dense, fine denticulae, extending onto oblique distal margin (Fig. 3K, L). Gonopod small, lateral to lamellar process, subcylindrical, posteriorly directed; eversible portion unobserved.

Telson and cercopods as typical for genus. Cercopods margined with plumose setae.

***Allotype female*.** Body reddish brown in life (Fig. 1D). Head smooth (Figs 2E, 5A), first antenna length subequal to second antenna, apex with five or six aesthetascs (Fig. 5B). Second antenna (Figs 2E, 5C) subcylindrical, with scattered, filiform setae, apex tapering to point (Fig. 5D, E). Thoracopods as in male. Thoracic segments X and XI with dorsolateral conical projections, apex spiniform (Figs 2F, 5G–I, L). Genital thoracic segments fused (Fig. 5L). Genital thoracic segments with single dorsolateral sensory seta surrounded by four or five ridges (Fig. 5I–K). Brood pouch extending to abdominal segment II (Fig. 5G), width subequal to thorax (Fig. 5L). Telson and cercopods as in male.

***Egg.*** Subspherical (Fig. 5N), diameter ~250 μm, with dense ridges ~50 μm wide, rounded in cross section. Ridges lacking spines, rarely closing to form polygons. Ridges separated by deep grooves ≤10 μm wide. Ridges rough, with dense, regularly distributed pores (Fig. 5O).

#### Etymology.

The specific epithet “*shangrilaensis*” refers to the species being from Shangri-La County. The gender is feminine.

## ﻿Discussion

*Parartemiopsis* was erected by Rogers (2005) to accommodate the Mongolian *P.mongolica*; only two male specimens were known at the time. Shortly after publication, Rogers realised that *P.mongolica* was a junior synonym of the Russian *Chirocephaluslongicornis* (Smirnov, 1930) (Rogers 2006), which was first described in the now unrecognised *Pristicephalus* Daday, 1910 and then moved to *Chirocephalus* (Brtek, 1967). However, the female was never properly described, although mentioned briefly by Smirnov (1930).

The males of both *Parartemiopsis* species share the basic form of the gonopods and the second antennae. Both share the anterior triangular projection on the distal antennomere. *Parartemiopsisshangrilaensis* sp. nov. is separated from *P.longicornis* by the form of the male second antenna. The antennal appendage is lobiform in *P.longicornis* and lamelliform in *P.shangrilaensis* sp. nov. The proximal antennomere lateral surface bears a large, medial, hemispherical bulge and a subdistal lobe in *P.longicornis*, which are absent in *P.shangrilaensis* sp. nov. The distal antennomere is straight in *P.longicornis* but curves medially in both the basal third and subapically in *P.shangrilaensis* sp. nov. In *P.shangrilaensis* sp. nov. the distal antennomere bears a basomedial, spiny, hemispherical protrusion in which is absent in *P.longicornis*. The form of the gonopod lamella also differs: it is subrectangular, with the length ~2× the length of the gonopod rigid base in *P.shangrilaensis* sp. nov., but triangular and subequal in length in *P.longicornis*. Furthermore, the medial spinulae row is elevated on an arcuate carina in *P.shangrilaensis* sp. nov., whereas this row is flat in *P.longicornis*. The female *P.longicornis* is incompletely known and in need of redescription according to modern standards. However, it appears from Smirnov’s (1930) description that *P.longicornis* has paired dorsolateral projections on thoracic segments V–X and small lateral spiniform projections on abdominal segments I–V. In contrast, *P.shangrilaensis* sp. nov. bears paired dorsal spiniform projections only on thoracic segments X and XI, with the abdomen inerm.

There is no information on the egg morphology of *P.longicornis*.

During fieldwork in 2012, the type locality was dominated by macrophytes, the water temperature was 22.1 °C, with a surface area of approximately 40 m^2^, and the depth was 0.3 m.

Previously, *Parartemiopsis* was only known from two locations, one in eastern Mongolia (Rogers 2005) and the other near Xingkai Lake or Khanka Lake (Smirnov 1930), near the border between China and Russia; both locations are in northeast Asia. The type locality of *P.shangrilaensis* sp. nov. is more than 2000 km from those sites. This new locality greatly enlarges the known distribution of the genus. More *Parartemiopsis* species may be found in future surveys in Asia.

### ﻿Key to *Parartemiopsis* Rogers, 2005

**Table:** 

1	Male antennal appendage lobiform, second antenna distal antennomere straight, lacking a medial, hemispherical, spiny bulge; female with dorsolateral conical projections on thoracic segments V–X and abdominal segments I–V	***Parartemiopsislongicornis* (Smirnov, 1930)**
–	Male antennal appendage lamellar, second antenna distal antennomere bent medially in proximal third and subapically, bearing a medial, hemispherical, spiny bulge; female with dorsolateral conical projections on thoracic segments X and XI only	***Parartemiopsisshangrilaensis* sp. nov.**

### ﻿Key to Chirocephalidae of China

*Chirocephalus* is in great need of revision (Rogers 2013). Recent attempts to erect or resurrect genera from *Chirocephalussensu lato* based on small genetic sampling from the genus (Weekers et al. 2002; Naganawa et al. 2020) have only created confusion. The genus, as it currently stands, is probably polyphyletic, but with 60+ species (Rogers 2013), phylogenetic studies on only a handful of species cannot accurately elucidate evolutionary relationships within the group. Until a larger number of species are analysed using standard, modern molecular and morphological techniques, the resurrection or establishment of new genera only creates more confusion. First and foremost, we need a definition of what *Chirocephalus* is. We need this before we can decide what is not *Chirocephalus*. Until then, the various proposed generic names remain available.

There are several records of *Chirocephalus* from China that are questionable. Chirocephalusspinicaudatusvar.chyzeri (Daday, 1890) was reported from Tibet by Chiang (1983), but his figures do not agree with the description of that taxon, which is only known from Slovakia and Romania (Belk and Brtek 1995). Similarly, *C.graziellae* Alonso & Naganawa, 2008 was reported from Tibet by Deng et al. (2021); again, the description provided of the Tibetan material disagrees with the original description and is insufficient to determine what species Deng et al. had. The material from Tibet of both populations needs to be examined and identified according to modern standards. Neither of these forms are included in the key below.

**Table:** 

1	Male distal antennomere anterior margin without a medial triangular projection; male genital segments not expanded, lacking a ventral transverse ridge; gonopod basal portions separated by their width; gonopod rigid basal portion bearing a medial, digitiform projection, with medial surface bearing spinulae; gonopod everted portion a short cone (*Chirocephalus*)	**2**
–	Male distal antennomere anterior margin with a medial triangular projection; male genital segments enlarged and chitinized with a transverse ridge along ventroposterior margin; gonopod basal portions separated by 3–4× their width; gonopod rigid basal portion bearing a large, ventromedial, subtriangular and broadly lamellar projection, with medioventral margin lined with spinulae; gonopod everted portion subcylindrical with apex digitiform	***Parartemiopsisshangrilaensis* sp. nov.**
2(1)	Apophyses subconical, cylindrical, or broadly expanded	**3**
–	Apophyses subglobular, spherical	***Chirocephalusmongolianus* Uéno, 1940**
3(2)	Second antennae distal antennomere with a basal projection(s)	**4**
–	Second antennae distal antennomere without a basal projection	***Chirocephaluswangi* Hsü, 1933**
4(3)	Second antenna distal antennomere nearly straight	***Chirocephalusnankinensis* (Shen, 1933)**
–	Second antenna distal antennomere bending medially nearly 90º	***Chirocephalussinensis* Thiele, 1907**

## Supplementary Material

XML Treatment for Parartemiopsis

XML Treatment for Parartemiopsis shangrilaensis

## ﻿Additional information

### Author ORCIDs

Shu-Sen Shu https://orcid.org/0000-0001-6476-004X

Xiao-Yong Chen https://orcid.org/0000-0002-0924-5560

D. Christopher Rogers https://orcid.org/0000-0003-3335-7287

Laorsri Sanoamuang https://orcid.org/0000-0003-0377-1225

### Data availability

All of the data that support the findings of this study are available in the main text.
